# Materials and structures used in meniscus repair and regeneration: a review

**DOI:** 10.1051/bmdcn/2019090102

**Published:** 2019-02-22

**Authors:** Ketankumar Vadodaria, Abhilash Kulkarni, E Santhini, Prakash Vasudevan

**Affiliations:** 1 Centre of Excellence for Medical Textiles, The South India Textile Research Association Coimbatore Tamilnadu India

**Keywords:** Meniscus, Biomaterial, Regeneration, Scaffold, Textile, Tissue engineering

## Abstract

Meniscus is a vital functional unit in knee joint. It acts as a lubricating structure, a nutrient transporting structure, as well as shock absorber during jumping, twisting and running and offers stability within the knee joint. It helps in load distribution, in bearing the tensile hoop stresses and balancing by providing a cushion effect between hard surfaces of two bones. Meniscus may be injured in sports, dancing, accident or any over stressed condition. Any meniscal lesion can lead to a gradual development of osteoarthritis or erosion of bone contact surface due to disturbed load and contact stress distribution caused by injury/pain. Once injured, the possibilities of self-repair are rare in avascular region of meniscus, due to lack of blood supply in avascular region. Meniscus has vascular and avascular regions in structure. Majority of the meniscus parts turn avascular with increase in age.

Purpose of this review is to highlight advances in meniscus repair with special focus on tissue engineering using textile/fiber based scaffolds, as well as the recent technical advances in scaffolds for meniscus recon- struction/ regeneration treatment.

**Abbreviations**
PCL: Poly caprolactone
DNA: Deoxynucleotide acid
PU: Poly urethane
UHMWPE: Ultrahigh molecular weight polyethylene
PGA: Poly glycolic acid
PVA: Polyvinyl alchohol
GAG: Glucosaminoglycans
TGF-pi: Transforming growth factor
PLLA: Poly-L-Lactic acid
bFGF: Basic fibroblast growth factor
IGF: Insulin Growth Factor
BMP: Bone morphogenetic proteins
ECM: xtracellular Matrix

## 1. Introduction

**Fig. 1:**
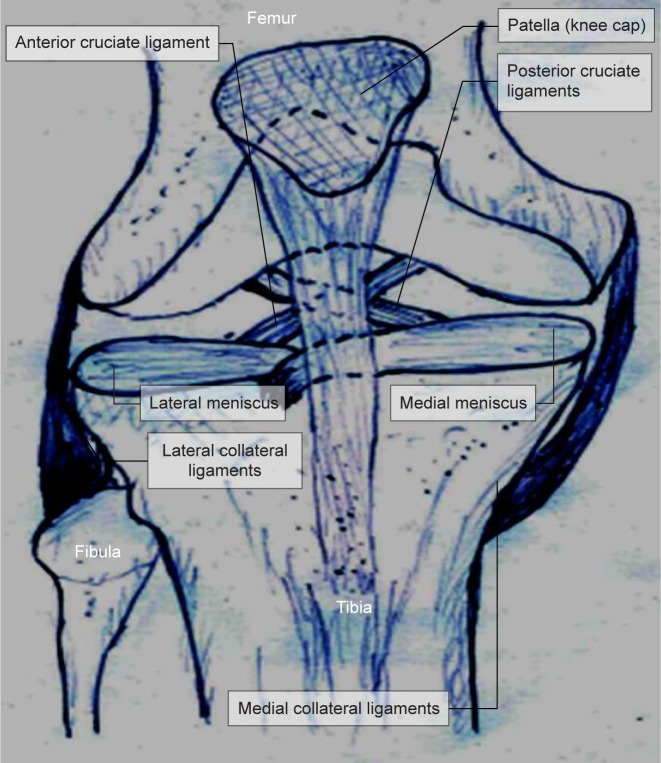
Schematic diagram of Anatomy of meniscus.

The knee joints are made up of bones (thigh bone, shin bone and knee cap), ligaments (joining tissues) and cartilage (meniscus) (Fig. 1). In the knee joint soft tissues (Menisci, tendons, ligaments, muscles) play a very important role of joining the femoral, tibia, fibula and the patella (knee cap). The meniscus are a pair of “C” shaped glossy white fibrocartilages in each of the knee joints [1]. The Greek word meniskos is the mother of word menisci - meaning “crescent” - moon shaped [2]. The sickle shaped meniscus is shaped to confirm the space between the femoral condyle (thigh bone) and tibia plateau (shin bone). There are menisci tissues on each side- (the medial and the lateral) of the knee.

Meniscus gives stability and support to the knee joint. Meniscus helps to balance a person when the knee joint is over-bent, over loaded, over twisted or over stretched. The semilunar shaped meniscus transfers the total body weight, compressive force, shear stress, circumferentially directed forces, tensile stress, etc to the lower leg every time a person walks, runs or jumps. The wedge shaped meniscus acts as a shock absorber when subjected under various forces and stresses such as shear, tensile and compressive. The physiological function of the meniscus are load bearing, load transmission / distribution, stability, shock absorption, lubrication, pressure resistance, joint filler (adjustment to tibia surfaces and modulation of femoral condoyle shape), sensory, proprioception and distribution of synovial fluid. They also assist in different motions and prevent synovial impingement. Meniscus also nourishes the articular cartilage, connective tissues for movable joints. In short, it provides a smooth, lubricated surface for articulation and facilitates load transmission with low friction co-efficient [1, 3–5].

## 2. Anatomy of the meniscus

**Fig. 2:**
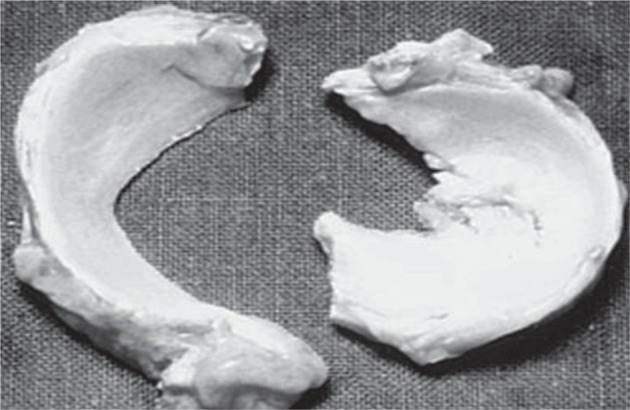
The medial and lateral meniscus removed after knee joint. [70].

The size of the meniscus is correlated with sex, height, weight, bone area and body mass index by many researchers [2, 3, 6, 7]. The meniscus cross sectional shape is triangular, which confirms the shape of tibia plateaus. It is thick, convex at outer rim, thin, and concave at the inner rim [2]. The medial meniscus is situated inside towards the leg and the lateral meniscus is situated at outside *i.e.* away from the leg. Approximately 51% to 74% of tibia area is covered medially by medial meniscus. It is crescent shaped with approximately 40.5 mm to 45.5 mm long, 27 mm wide and 35 mm diameter [10]. Lateral refers to side; approx. 75%-95% of tibia is laterally covered by lateral meniscus. It is more circular in shape with approximately 32.4 mm to 35.7 mm length and 26.6 mm to 29.3 mm width (Fig. 2) [1, 2, 8].

**Fig. 3:**
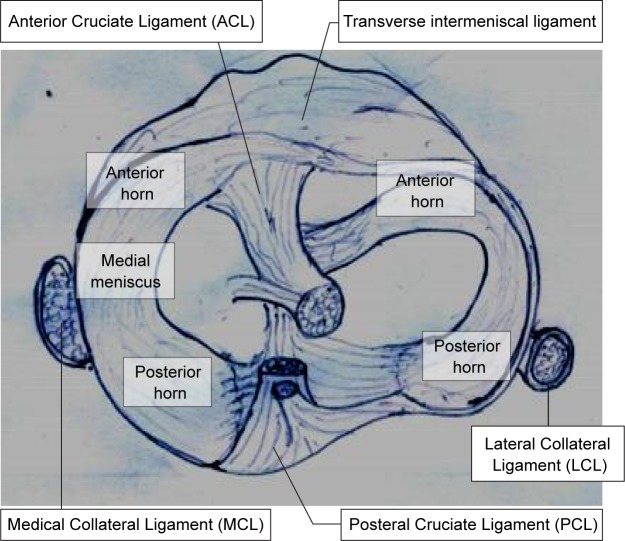
Schematic diagram of meniscus anatomy.

The main stabilizing ligaments in knee joints are the medial collateral ligaments, the transverse ligaments and the meniscofemoral ligaments (Fig. 3). Meniscus is attached medially to medial collateral ligament and laterally to the menisco-femoral ligament as well as to the transverse ligaments and joint capsule [9]. The transverse ligament connects both menisci. They are anchored to the tibia *via* anterior horn and the posterior horn [2]. Mainly meniscal intersectional ligaments are anchored to tibia bone *via* the anterior horn and the posterior horn. The lateral meniscus is attached to femur *via* meniscofemoral ligaments (Humphrey (anterior) and Wrisberg (posterior) ligaments) and bone near the insertion site of the posterior cruciate ligament in the medial femoral condyle [1]. The anterior (front) meniscus hom is attached to the tibia and connected to the anterior cruciate and transverse ligament. Transverse ligament joins two menisci together. The anterior (front) medial meniscus hom is attached to the tibia and connected to the anterior cruciate and transverse ligament [10].

## 3. Vascular anatomy

**Fig. 4:**
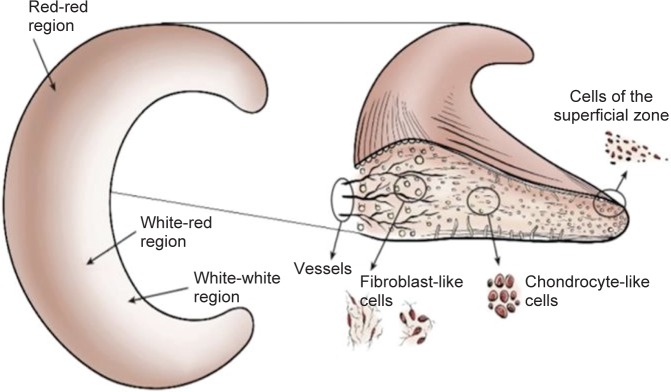
Left-Regional variations in vascularization and cell populations of the meniscus; Right- type of cell population. [1]

The vascularity / blood supply of the meniscus diminishes gradually with age. The new born child’s meniscus are fully vascularized, while in the adult the vascularity diminishes and remains limited to the outer periphery only. With progressing age, the majority of vascularity in meniscus proportion disappears. The lack of blood supply means lack of nutrients, oxygen *etc.* Once it is damaged, it has limited repair capacity in the avascular region of meniscus. Only 10% to 30% of total meniscus can be found with blood vessels in 10 years old child [9]. In the case of an adult, 10% to 25% of the outer lateral meniscus periphery remains vascular. The meniscus can be divided into three distinct regions based on vascularity - the highly vascularized (red) outer periphery region, the avascular (white) inner region and red-white joining transition region with property of both red and white regions. The self-repair capacity of different meniscus regions differs directly with the presence of the blood vessels. Hence, it becomes necessary to remove and repair the meniscus in majority of the meniscus lesion cases (Fig. 4) [1].

## 4. Composition and structure

**Fig. 5:**
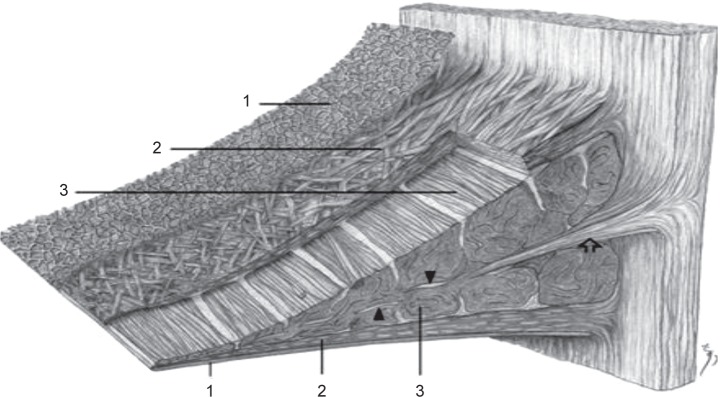
Synoptic drawing. Scanning electron microscopy reveals three distinct layers in the meniscus cross section: (1) The superficial network. (2) Lamellar layer (3) Central main layer. [71]

**Fig. 6:**
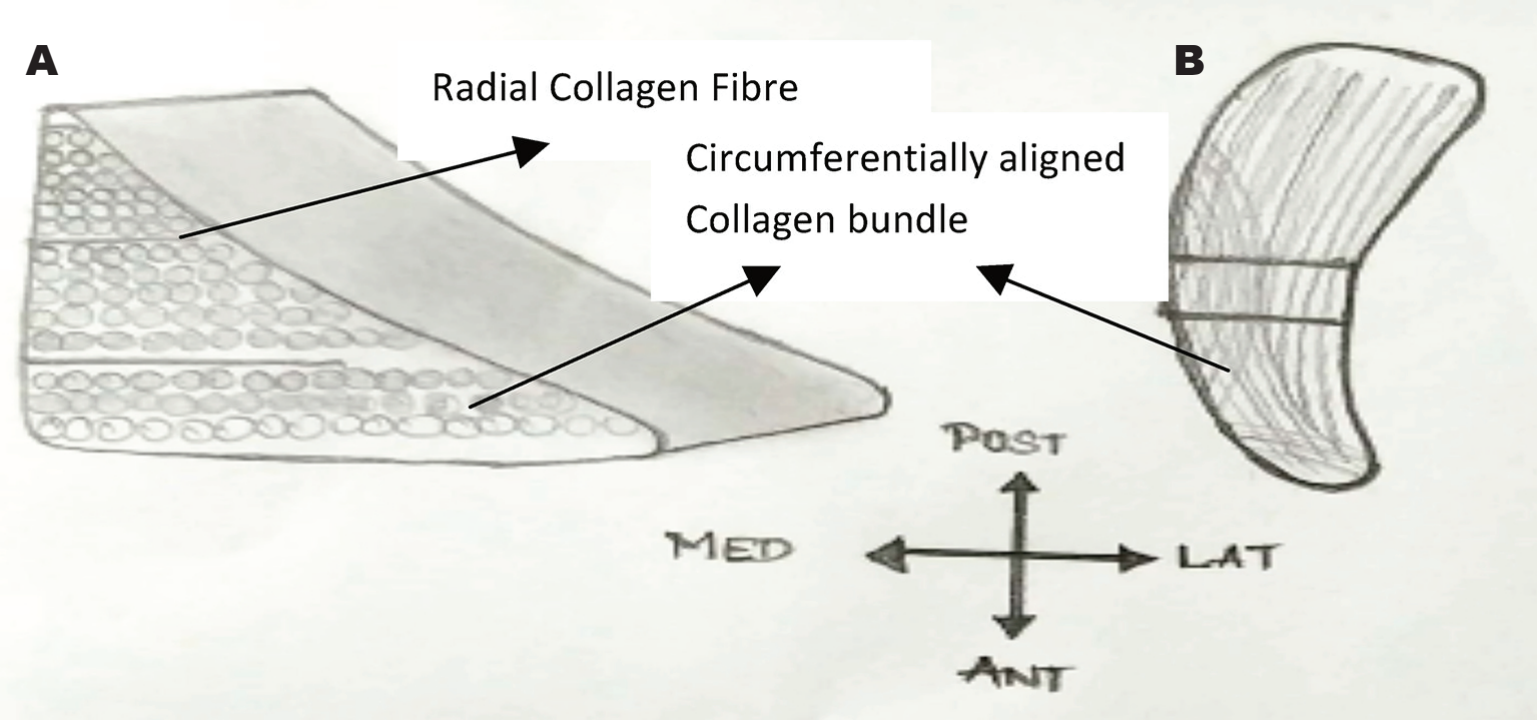
Illustration of a meniscus showing (A) a wedge-like cross-section displaying a simplified collagen fiber organization, with the majority of fiber bundles in the circumferential direction with occasional radial ‘‘tie’’ fibers and (B) the generalized anatomic macrostructure. [63]

Meniscus compositions may vary depending on various factors such as meniscus region (red, white or red- white), gender, age, type of injury and other pathological conditions [1]. Meniscus contains heterogeneous cell populations with rounded or oval shaped chondrocyte like cells in inner two third portion, the outer one- third of the meniscus is populated by spindle shaped fibroblast-like cells [9, 11]. The cellular meniscal components also include fibrochondrocytes interspersed within the extracellular matrix. Fibrochondrocytes display the properties of both fibroblasts and chondrocytes (Fig. 6) [10]. Human meniscus is composed of approximately 65% to 85% water and 30% organic matter. The water content helps in providing cushion / lubrication effect as well as in transferring nutrients [12]. Usually dry matter composed of mainly collagen (75%), Glycosaminoglycans (GAGs) (17%) [13], DNA (up to 2%), elastin (less than 1%), adhesive glycoproteins (less than 1%) in meniscus matrix. Collagen type I is the dominating collagen (90% dry weight) [9, 14]. Type II, III, V, VI also exist in minor proportions [2, 9, 15]. Collagen contributes towards meniscus strength. Collagen fibers run circumferentially parallel to the peripheral border [8]. Microscopically, three types of collagen fiber layers are found in the meniscus tissue as shown in Fig. 5 and 6 to bear loads. The superficial layer consists of meshwork; second layer under the superficial layer is made up of lamella like collagen fibrils. The larger fibers run circumferentially parallel in the middle layer. In the deep layer, the bundles of aligned collagen fibers parallel to the periphery can be found [9, 10]. The proteoglycans enables the meniscus to absorb the water. The water helps meniscus to bear forces, compression, etc by providing lubrication / cushion effect and nutrient transport [2, 12, 15]. Smaller proportion of elastin helps in shape recovery after load [10].

## 5. Meniscus repair and redevelopment

Annually, the treatment of meniscus related surgical cases alone are over 4, 00, 000 in Europe and over million in USA at an estimated cost of $4 billion [9, 16].

Meniscus tear can occur due to over twisting / bending of knee joint due to sports, accidents, brittleness in aged people, or any other reason. The symptoms vary from i) severe joint pain, swelling and renders around knee area, iii) even pain sensitive upon touched, iv) popping or creaking sound, while walking, v) locking of knee, vi) difficulty in straightening of leg vii) instability in standing position and viii) misalignment of limb [4]. Another problem associated with meniscus is the Total menisctomy, which can lead to osteoarthritis due to cartilage degeneration [1].

The healing of meniscus damage depends on the region of the damage. Any damage in vascularized region (red) can often be healed by stitching. Whereas, healing of any tear in the inner avascular region (white) may be trickier due to lack of blood flow [17]. In the case of meniscus injury/damage, the options left are meniscectomy, repair and transplantation. It is well documented that total meniscectomy leads to early osteoarthritis due to wear, tear, misalignments *etc.* [1, 14]. Hence, total or subtotal meniscectomy is only preferred in the case of non-reparable damage over partial meniscectomy and meniscus - repairing. When a piece of meniscus is removed, over a period of time it may disintegrate or degenerate because of axial deviation, overweight or overwork. In that case, some objects are needed to fill in the gap *(i.e.* meniscus regeneration).

Different approaches are adapted for meniscus repair/regeneration depending on the type of lesion and type of region - vascular or avascular [18]. The primary aim is to keep the meniscus tissues intact while attaching meniscus during the surgery [1, 19, 20]. Below is the list of available meniscus treatment approaches.

- Meniscal tear repairs: the torn part of meniscus is sutured to repair
- Partial meniscectomy
- Gene therapy and growth factor
- Intra articular cell delivery
- Meniscal Replacement: Allograft /Autograft transplantation
- Tissue Engineering: Meniscal Scaffold / Implant

In spite of all the above methods, there is no universal method available to promise fully established, regenerated, functional and long-lasting meniscal tissues. The meniscus treatment depends upon several factors such as the type of tear, tear pattern, geometry, site vascularity, size, stability, tissue viability, tissue quality, associated pathology, surgery, advancing age, degeneration pattern, lack of tissue viability (attenuation and degeneration), patient’s medical condition and patient’s preference after counseling [21].

### 5.1. Autograft and allograft

There are two types of meniscal replacement/reconstruction: Autograft and Allograft. Autograft is a patient’s own tissue and in contrary Allograft is a tissue from a donor person to repair the damaged meniscus. Kohn *et al.,* replaced sheep meniscus with a fat pad autograft [22, 23]. However, they concluded with the nonsuitability of fat as a meniscus substitute. Autograft derived from various sources such as fat pad, tendon, cartilage, periosteum, synovial flap and perichondrium, did not show encouraging results [4].

The allograft transplantation for meniscus is considered as a symptomatic choice for treatment. Allograft can be freeze-dried, fresh, fresh-frozen and cryopreserved. The advantage and disadvantage of the above preservation methods on the final properties of allograft are described by Sgaglione [21]. According to Verdonk *et al.,* viability of the cells drastically dropped when the allograft is deep frozen considering to cryopreservation technique which retains about 10-30% viable fibroblast cells. With wide variation in results, some allograft transplantation studies mentioned benefits such as pain reduction, improved functionality of knee joint at short, medium and long term follow-up, improvement in level of disability, protection of articular cartilage and prevention of arthrosis [4, 20, 24, 25]. Allograft treatment is reported such as graft processing, and graft conservation. Various factors such as graft effect on biochemical / chemical properties, possibility of immunological reaction, immune rejection, structural and property variation among different implants, disease transmission, perfect graft size and attachment, poor mechanical performance, fixed pore geometry, shrinkage, availability, cost, difficulty in logistics, cost of preservation and implant, effect of sterilization and preservation on biomechanical performance, procedure to fix the allograft, mismatch of allograft, *etc.* play a significant role in the rejection or the cons of the allograft treatment [2, 4, 14, 19, 26].

### 5.2. Gene therapy and growth factors

Growth factors are ‘signalling protein molecules’ in tissue/ cell culture. They stimulate the synthesis as well as inhibit extra cellular matrix (ECM) degradation. They bind to their respective receptors in the cell to promote cell differentiation and proliferation, cell migration, and matrix synthesis [18, 27, 28]. Meniscus treatment using various growth factors showed benefits such as accelerated proliferation; superior cell numbers, migration and alignment; increased production of collagen and proteoglycans; healing of tear in avascular region; blood vessel formation *etc*. Transforming growth factor (TGF-pi) stimulates synthesis of specific proteoglans, ECM and collagen type II. Basic fibroblast growth factor (bFGF) can help in proliferation and production of meniscus cell and new extra cellular matrix [27]. Other growth factors are also studied on its effect for meniscus regeneration such as Fibroblast growth factor-2 (FGF - 2), Insulin growth factor (IGFs), Vascular endothelial growth factor (VEGF), Bone morphogenetic proteins (BMPs) [19, 27]. Liu *et al.,* provides a summary of various studies reporting the effect of different growth factors on meniscus.

Gene therapy has displayed a tremendous potential for menis- cal repair in both *in vitro* and *in vivo* tests [29–31]. It is found that localized delivery of gene can induce healing with targeted localized high concentration at the site of interest without harming other cells, which will accelerate the process of repair. *in vivo* and *in vitro* gene transfer showed potential in meniscal repair. In gene therapy, there are a number of parameters and limitations to be considered for its use in mensical repair. These parameters are modality of transduction, ideal gene combination, efficient cell type in the case of transferred cells, identifying appropriate genes, gene therapy approach / method, optimization of gene delivery system, optimization of level and duration of gene expression etc [17, 31]. Hikada *et al.,* treated tissue-engineered meniscus with Hepatocyte growth factor gene (AdHGF) using adenovirus vector encoding for vascularization [32]. The result showed formation of new blood vessels *i.e.* 2.5 to 4 times than control.

### 5.3. Intra-articular cell delivery

The treatment may include delivering the cell source to the defective site. The intra articular injection of progenitor cells is another attractive option for meniscus treatment. Few studies have shown active participation of injected cells, successful cell survival, and cell activity in the knee joint. Despite promising results, this approach needs more large-scale animal studies to be considered as treatment option in future [19, 20, 33, 34].

## 6. Materials and structures used in meniscus repair and regeneration

### 6.1 Scaffolds and cell seeding

Scaffold based tissue engineering for knee meniscus reconstruction / regeneration can offer many advantages over other techniques such as:

- Control over custom made shape designs / porosity / structures using different production techniques
- Flexibility in designing scaffold properties such as mechanical performance / biodegradable characteristics by changing material combinations or structures;
- Easy availability
- Cost
- Reproducibility and Repeatability.

Textile based materials, solvent casting, particulate leaching, membrane lamination, melt molding, 3D printing, phase separation, *etc.* are few of the scaffold fabrication techniques.

Laurencin *et al*., defined tissue engineering as ‘‘the application of biological, chemical and engineering principles towards the repair, restoration or regeneration of living tissues using biomaterials, cells, and factors alone or in combination” [35]. Scaffold structure, scaffold material (synthetic or natural), type of cell seeded and specific stimuli *(e.g.* growth factor, gene therapy) can be considered as important factors in tissue engineering, with many challenges in tissue engineering such as producing uniform pore size, distribution and inter connectivity [16].

Scaffold seeded with specific cell type based on the requirement can be used for engineered tissue regeneration similar to native cells [1]. There are different types of cells, which can be considered for meniscus regeneration [1] - Autologous cells, Allogeneic and xenogeneic cells, stem cells (Human embryonic or Adult) or combination of the above. Scaffold design must be done considering several factors such as the macro - micro level, external geometry including factors such as the bioactivity (inherent to human system), and mechanics similar to meniscus. Ideal scaffold material and structure must have the following characteristics [34, 36]:

- Be non-toxic, biocompatible, non-carcinogenic
- Easily sterilisable
- Allow / guide cells, allow differentiation / proliferation, migration, attachment, growth by providing optimum pore density, pore size, pore distribution, pore inter-connectivity, etc.
- Correct fiber orientation to guide cell orientation and support
- Optimum surface chemistry / properties cell attachment and proliferation
- Biocompatibility to avoid rejection
- Desired biodegradability
- Desired mechanical performance (strength and destruction)
- Desired permeability of macromolecules and nutrients.
- Provide path to eliminate waste molecules
- Ability to engineer implant shape as well as *in vivo* stability.

The meniscus tissue can be substituted by a synthetic polymeric scaffold [37, 38] tissue derived materials or hybrid / composites of all the above [9, 19, 21, 39]. The menisci cells consist of fibroblasts and chondrocytes (called fibrochondrocytes). Scaffolds produced form synthetic material can exhibit limitations in terms of high rejection, unpredicted degradation or swelling and every scaffold implant requires repeated study for a long time before considering them as a scaffold for implantation [9].

Some of the challenges in meniscus implant include [2]:

- Fixation of the graft to tibia and joint capsule to prevent implant extrusion
- Engineering of structure to match the compressive and tensile properties
- Selection of the surface characteristics to minimize chon- dreal damage to the femur and tibia
- Mimic tribology of the native meniscus
- Reproducibility into 3 dimensional structure
- Degradation and degraded product should be nontoxic.
- Suture resistance to tear
- Comparable strength to that of meniscus cartilage.

Porous scaffolds are suited for tissue engineering. The pore size and its interconnectivity, 3-D surface environments, *etc.* are conducive to promote cell-to-cell contact. Scaffolds are classified into two fields: Natural and synthetic. The natural scaffolds are primarily from a natural component of tissue derived matrix such as collagen, perichondrial tissue and hyaluroan. Synthetic scaffolds are made up of synthetic polymeric materials such as polyurethane, polycaprolactone, Polylactic acid, Polyglycolic acid and Polylatic-co-glycolic acid.

**Fig. 7:**
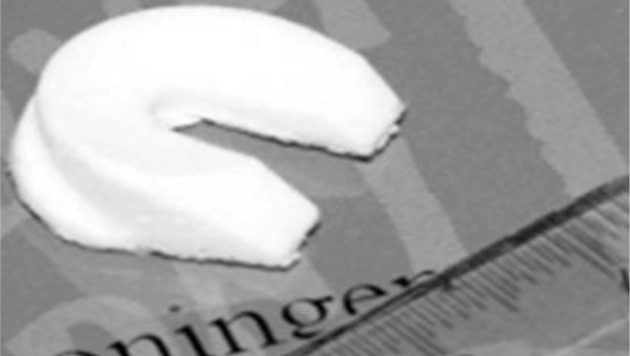
Meniscus shaped scaffold. [40]

**Fig. 8:**
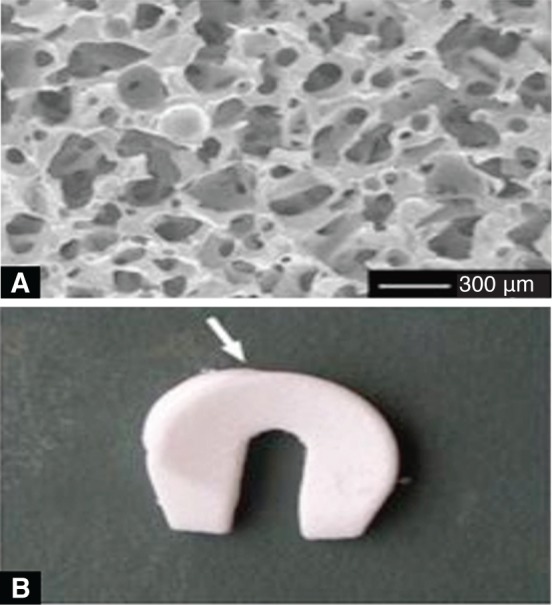
(A) Scanning electron microscopy image of a cross section of the polycaprolacton-based polyurethane (PCLPU) implant. (B) Porous PCLPU meniscus implant provided with an interstice for the popliteus tendon (arrow). [41]

As shown in Fig. 7, Heijkants *et al.,* cut meniscus shape from a block of foam [40]. He synthesized a liner biomedical segmented polyurethane (PU) with polycaprolactum (PCL) as soft segment and 1, 4-butanediisocyanate and chain extended with butanediol as hard segment as shown in Fig. 8. This material showed excellent mechanical properties. The porous foam with interconnected pores was prepared by salt leaching. The foam was cut into menisci shape and implanted into the knee of a dog. The foam structure allowed tissue growth with comparable compression behavior. At the same time, in a similar study by Hannink *et al.,* found discouraging results [41]. The study concluded with the findings that the above porous implant is not suitable for meniscus replacement due to cartilage damage and degradation compared with the native.

Porous scaffold based on PCL and hyaluronic acid (PCL/ HYAFF) showed vascularized tissue growth in sheep [42]. Despite encouraging results, the overall results were not encouraging; the implant could not protect the knee joint from arthritis. Using similar salt leaching technique, Chairi developed a meniscus like device by lamination technique using moulds from Hyaluronic acid and PCL derived biomaterial [39]. The salt was used in the ratio of 1/10 with biomaterial to ensure inter connected pores. In this case, new tissue formed all over the implant surface bonding to the capsule in core zone of implant, new collagen fibers were formed between biomaterial and original meniscus, growth of blood vessels from superficial to central area of the biomaterial, *etc.* suggested promising results. The implants remained in position, retained their shape and maintained adequate mechanical properties. However, defects such as compression of implants and extrusion were observed.

**Fig. 9:**
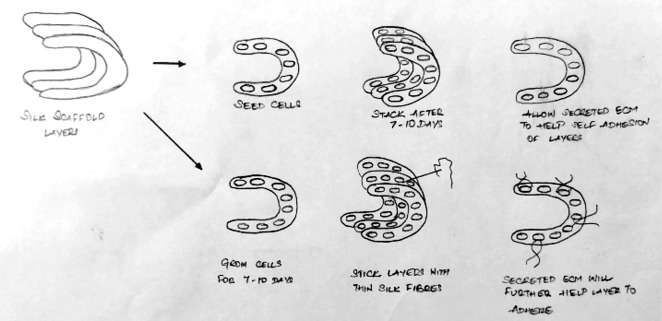
Silk based reconstruct for meniscus graft applications.

Mandal *et al.,* proposed 3 layered silk meniscal scaffold (Fig. 9) seeded with human fibroblasts (outside) and chondrocytes (inside) in a similar system to mimic native meniscus native architecture [9]. The scaffold showed promising mechanical properties, increase in GAG proteoglycans production as well as directed tissue growth with aligned ECM deposition. Mueller *et al.,* examined use of porous collagen I and II - GAG copolymer scaffold seeded with calf meniscus cells [43]. The cells showed early proliferation and more uniform cell distribution. Authors concluded that the higher GAG synthesis with collagen specific behavior and poor mechanical strength demand further investigation.

Yan *et al.,* described a methodology to fabricate porous silk fibroin scaffold by salt leaching and freeze drying for meniscus tissue engineering [44]. Scaffold exhibited good water uptake capacity, porosity as well as pore interconnectivity, the porosity decreased with higher silk fibroin concentration in solution. The author also claimed retention of original mechanical properties of silk fibroin scaffold even after 30 days of immersion in an isotonic saline solution prepared for scaffold degradation.

### 6.2 Textile / fiber based scaffold

The textile based (nano /micro/macro fibers and fibrous structures, yarn / filament, woven, knitted, non woven, braided, fiber- reinforced composites, *etc.)* scaffolds present many benefits over other scaffold structures such as they offer high surface area to volume ratio, highly inter connected porous structure, possibility of 2D or 3D structure, easy producibility *etc.* However, structural stability is one of the cons found in the textile-based scaffolds, which can be overcome by fiber-reinforced composite (FRC) [45, 46]. Recently, lot of work is done on fiber reinforced composites to aid in the repair of the meniscus. Holloway *et al.,* and El-Amin *et al.,* developed meniscus implant using ultrahigh molecular weight polyethylene (UHMWPE) fibers reinforced in polyvinyl alcohol [47–49]. Fiber-matrix interfacial properties and mechanical performance were studied by varying different spacing between fibers. The scaffold showed comparable tensile properties to native meniscus and its ability to manufacture meniscus like geometry. The authors concluded that there was a need of further study to understand the different properties (geometry, material *etc.)* towards the development of functional meniscus. Gunja *et al.,* injected poly-L-lactic acid (PLLA) nonwoven scaffold with meniscus cells (MCs) and articular chondrocytes (ACs) [11]. The scaffold showed functional tissue engineered meniscus constructs with biochemical and biomechanical properties. The authors claimed that GAG, collagen level can be achieved by varying the concentration of MCs and ACs co-culture ratios. In another experiment, Gunja *et al.,* cultured meniscus cells on cylindrical nonwoven poly-L-lactic acid scaffolds in normoxic (21% oxygen) or hypoxic (2% oxygen) conditions in the presence or absence of basic fibroblast growth factor (bFGF) [11]. The authors claimed significant enhancement in GAG, collagen II content and cell number/construct in groups exposed to hypoxia and bFGF compared to the controls.

Neves *et al.,* studied bioartificial meniscal cartilage construction from knitted polyethylene terepthalate fabric using fibrochon- dryocytes from the menisci of 6-month-old sheep and found out that scaffold geometry has significant impact on the properties of these scaffolds [51]. Chen *et al.* developed scaffolds consisting of web like collagen micro sponge formed upon knitted PLGA fabric [52]. The knitted fabric provided the scaffold with mechanical integrity, while the type I collagen micro sponge filled the large pores of the fabric and facilitated uniform cell distribution, cell attachment and tissue formation. After being implanted in nude mice for a period of 8 weeks, bovine chondrocytes seeded within the composite scaffold were shown to have maintained their natural morphology while producing collagen type II and ECM proteins.

**Fig. 10:**
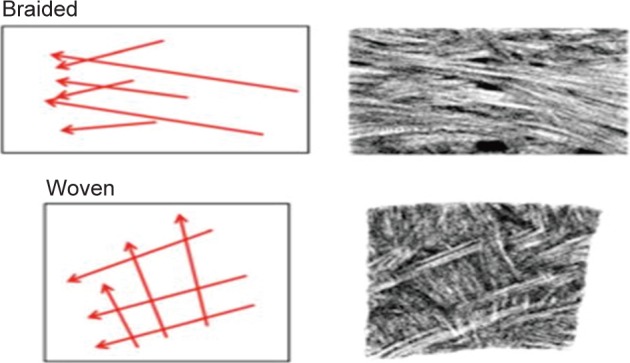
Schematic representation (left) of braided and woven fascicle organizations with associated sections from meniscal samples illustrating these arrangements (right). [53]

**Fig. 11:**
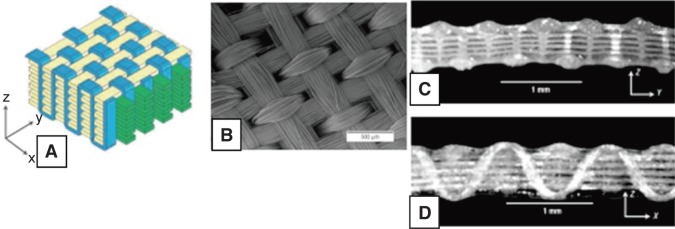
Woven structures (A) 3-D Illustration of orthogonal weave structure by interlocking of multiple layers, (B) Scanning electron microscope image of X-Y plane surface view (C) Y-Z plane cross section view and (D) X-Z plane cross section view. [54]

**Fig. 12:**
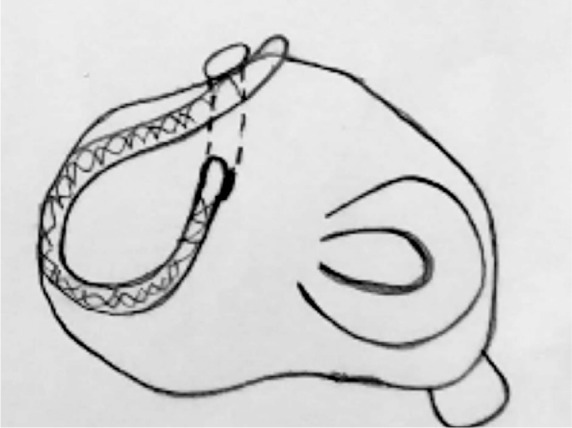
Meniscus Implants-Schematic view of the position of the prosthetic meniscus on the left tibia 1 plateau. [56]

Andrew *et al.,* studied meniscal complex collagenous fibrous fascicle arrangement in circumferential, oblique and radial direction using optical projection tomography [53]. The author identified woven or braided kind of fascicle arrangements in the observation (Fig. 10), which may help in load bearing and fracture toughness of meniscus. Makris *et al.,* concluded that studies on woven structure by Moutos *et al.,* for articular cartilage engineering could be useful to develop meniscus type anisotropic structure [54]. Moutos *et al.,* developed an anisotropic fiber-reinforced structure from 3D weaving of PLGA (Fig. 11) and PCL fibers consolidated with chondriocyte- hydrogel mixture into cartilage tissue construct showing mechanical properties (tensile, compressive, shear) comparable to the native tissue [54, 55]. Wood *et al.,* developed meniscus with little meniscus regeneration activity from concentrically stacked hoops of carbon fiber unsheathed by high tenacity polyester fiber extended by two braided threads for transosseous anchorage at horns (Fig. 12) [56]. Marsano *et al.,* developed nonwoven meshes of esterified hyaluronan seeded with chondriocytes to culture meniscus tissue. The developed construct resembled some aspects of complex structure and function of the outer and inner zones of native meniscus [57].

Kang *et al.,* explored the possibility of regenerating whole rabbit meniscus by implanting meniscal cells on the scaffold fabricated from polyglycolic acid (PGA) reinforced with poly (lactic- co-glycolic acid) [58]. However, author claimed that further investigations were needed to establish mechanical property and long-term data. Balint *et al.,* developed fiber reinforced collagen scaffold from acid insoluble bovine dermal collagen reinforced by a network of defadable tyrosine derived polymer fibers, poly (desaminotyrosyl-tyrosine dodecyl ester dodecanoate) P(DTD DD) only to determine the tensile properties [59].

**Fig. 13:**
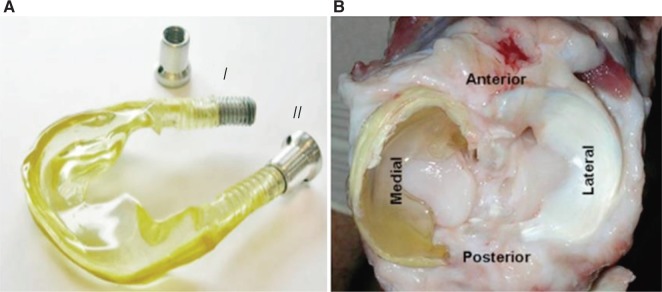
(A) Polycarbonate-urethane meniscal implant, with the stainless steel fixation bolt in the unfastened (i) and fastened state (ii), and (B) transverse view of an implant, in situ, in an exposed sheep cadaver joint. [60]

Kevlar reinforced polycarbonate-urethane has also been tried by Zur *et al.,* and Elsner *et al.,* implanted in a sheep as shown in Fig. 13 [60, 61]. The high modulus Kevlar fibers were reinforced circumferentially in the implant. Although the results showed mild cartilage degeneration, the implant to found so well anchored with no sign of any extrusion, migration or displacement from the implantation site. The material and structural properties did not register any change in properties.

Nonwoven mesh of PGA was and agarose hydro gel was cultured with fibrochondriocytes from rabbits. The PGA nonwovens mesh showed very encouraging results with 22 times higher sulfated glycoseaminoglycans and 3 times collagen presence compared to agarose hydro gel after 7 weeks [62].

### 6.3 Electrospinning

Apart from various textile structures, nanofibrous scaffold can be the ideal structure for meniscus tissue engineering. Properties of nanofibers such as high surface area to volume / mass ratio, porous structure, inter connected pores, cellular dimension of fibers, etc make them strong candidates for tissue engineering scaffolds. Electrospinning is the most versatile, simple and industrially viable method for nanofiber production.

Fisher *et al.,* fabricated circumferentially aligned scaffolds by changing the collection drum to the disc. The advantage of aligned nanofiber is that it can direct cell growth in the direction of fiber alignment [63]. The alignment dictates the cell growth direction. Baker *et al.,* concluded that mechanical performance of the mesenchymal stem cell can be improved by loading poly caprolactone (80 KDa) nanofibrous scaffold with cyclic loading compared to free swelling sample [64]. Baker *et al.,* seeded the above scaffold by meniscus-derived cells from surgical debris and observed a strong correlation between the amount of collagen deposition within the construct and the mechanical properties. Baker *et al.,* reported that aligned PCL nanofibers act as a micro pattern to direct tissue growth. They also concluded that mechanical performance of aligned scaffolds was better than the nonaligned scaffolds [66].

Lonescu *et al*., concluded that the growth factors (basic fibroblast growth factor (bFGF), transforming growth factor (TGT - B3)) and poly caprolactone nanofibrous scaffold showed comparable or superior integration compared to native tissue [29].

## 7. Commercial polymeric scaffolds

Two types of meniscus implants are successful.

1. Implant that provides structure and environment to encourage tissue regeneration (for *e.g.* CMI®, Actifit®, Fibrofix®). 2. Synthetic Permanent implants (for *e.g.* Nusurface), Menaflex® (by ReGen Biologics, USA) is the FDA approved collagen (derived from purified bovine Achilles tendons) meniscus implant (CMI^®^). Sponge like, gamma irradiated Menaflex^®^ is treated with hyaluronic acid, chondroitin sulphate, glycosaminoglycan and crosslinked with formaldehyde [2]. CMI® showed good to excellent clinical results without any adverse effect [67]. CMI® cannot be used as a full implant. Sgaglione *et al.,* reported replacement of collagen with a cell similar to meniscofibrochondrocytes as well as tissue regeneration and maturation similar to native meniscus cartilage [Kohn *et al.,* 1993]. The literature also reported CMI shrinkage over a period of time. Actifit^®^ (Actifit^®^, Orteq Ltd, LONDON, UK) is another commercially available porous meniscus implant, which is composed of polyurethane - polycapro- lactone (PU - PCL). Actifit has higher mechanical strength and much slower degradation rate than CMI® [17, 19, 68]. Fibrofix® is another silk fibroin, large polypeptides based scaffold developed by Oxford biomaterials. Nusurface is the first synthetic implant for total meniscus replacement. Nusurface^®^ is developed in Israel and still under clinical trials. It is made from polyethylene reinforced polycarbonate urethane (PCU). TRAMMPOLIN (Total Replacement of Meniscus with Minimally Invasive Polymer Implant) is the Dutch consortium with a goal to develop nonresorb- able meniscus substitute [69]. Different experts from material science, tribology, biomechanical engineering, biology, surgery, *etc*. are working to study different aspects for the development of TRAMMPOLIN meniscus [67].

## 8. Conclusion

Meniscus damage can occur due to over loading, over twisting, over stretching, over bending, *etc*. Meniscus lesion is one of the common injuries in sports, dancing, and accidents and inclined mainly due to ageing. Another problem apart from the above is the cartilage degeneration, which leads to osteoarthritis. The meniscus treatment becomes trickier because of lack of blood supply in majority of the structures. This review has covered all latest available approaches and pathways leading to repairing of the meniscus, its reconstruction and regeneration in the event of damage. Among different approaches, tissue engineering is leading the way and can be considered as one of the fastest growing fields. Lot of advancement has been done in the field of biomaterials that can be synchronised with tissue engineering to solve the issues associated with meniscus repairs. Despite latest developments in tissue engineering, lot of work must be focused on mimicking the biomechanical functionality of the meniscus. Considering the latest developments in biomaterials, all disciplines must be combined to mimic the meniscus properties to achieve the goal of me- niscal replacement with the healthcare costs taken into account. Tissue engineering by allograft / autograft has certain limitations such as chances of infection, rejection, availability, cost, preservation, *etc*. Because of all the above limitations, the tissue engineering research focus is towards synthetic or natural material based scaffolds. The easy availability, easy processability, ability to tailor scaffold properties and structure, *etc*. prove that synthetic material based scaffolds are more versatile for future tissue engineering approaches. The present review covered different materials and scaffold structures used for meniscus implant with a focus on textile based scaffolds. The review also covered commercially available partial or full meniscus replacements.

## References

[1] Makris EA , Hadidi P , Athanasiou KA . The knee meniscus: structure-function, pathophysiology, current repair techniques, and prospects for regeneration. Biomaterials. 2011; 32(30): 7411–31 21764438 10.1016/j.biomaterials.2011.06.037 PMC3161498

[2] Fox AJS , Wanivenhaus F , Burge AJ , Warren RF , Rodeo SA . The human meniscus: A review of anatomy, function, injury, and advances in treatment. Clin Anat. 2015; 28(2): 269–87 25125315 10.1002/ca.22456

[3] Rath E , Richmond JC . The menisci: basic science and advances in treatment. Br J Sports Med. 2000; 34: 252–7 10953895 10.1136/bjsm.34.4.252 PMC1724227

[4] Liu C , Toma IC , Mastrogiacomo M , Krettek C , Von Lewinski G , Jagodzinski M . Meniscus reconstruction: today’s achievements and premises for the future. Arch Orthop Trauma Surg. 2013; 133(1); 95–109 23076654 10.1007/s00402-012-1624-2

[5] Messner K , Gao J . The menisci of the knee joint. Anatomical and functional characteristics and a rationale for clinical treatment. J Anat. 1998; 193(2): 161–78 9827632 10.1046/j.1469-7580.1998.19320161.x PMC1467836

[6] Van Thiel GS , Verma N , Yanke A , Basu S , Farr J , Cole B . Meniscal allograft size can be predicted by height, weight, and gender. Arthroscopy. 2009; 25(7): 722–7 19560635 10.1016/j.arthro.2009.01.004

[7] Stone KR , Freyer A , Turek T , Walgenbach AW , Wadhwa S , Crues J . Meniscal sizing based on gender, height, and weight. Arthroscopy. 2007; 23(5): 503–8 17478281 10.1016/j.arthro.2006.12.025

[8] Fox AJS , Bedi A , Rodeo SA . The basic science of human knee menisci: structure, composition, and function. Sports Health. 2012; 4(4): 340–51 23016106 10.1177/1941738111429419 PMC3435920

[9] Mandal BB , Park SH , Gil ES , Kaplan DL . Multilayered silk scaffolds for meniscus tissue engineering. Biomaterials. 2011; 32(2): 639–51 20926132 10.1016/j.biomaterials.2010.08.115 PMC2991374

[10] Brindle T , Nyland J , Johnson DL . The Meniscus: Review of Basic Principles. J Athl Train. 2001; 36(2): 160–9 16558666 PMC155528

[11] Gunja NJ , Athanasiou KA . Effects of co-cultures of meniscus cells and articular chondrocytes on PLLA scaffolds. Biotechnol Bioeng. 2009; 103(4): 808–16 19274749 10.1002/bit.22301

[12] Lewis PB , McCarty LP , Kang RW , Cole BJ . Basic science and treatment options for articular cartilage injuries. J Orthop Sports Phys Ther. 2006; 36(10): 717–27 17063834 10.2519/jospt.2006.2175

[13] Herwig J , Egner E , Buddecke E . Chemical changes of human knee joint menisci in various stages of degeneration. Ann Rheum Dis 1984; 43(4): 635–40 6548109 10.1136/ard.43.4.635 PMC1001426

[14] Drengk A , Stürmer KM , Frosch K . Current Concepts in Meniscus Tissue Engineering. Curr Rheumatol Rev. 2008; 4(3): 1–6

[15] Hellio LGMP , Vignon E , Otterness IG , Hart DA . Early changes in lapine menisci during osteoarthritis development: Part I: cellular and matrix alterations. Osteoarthr. Cartil. 2001; 9(1): 56–64 11178948 10.1053/joca.2000.0350

[16] Belluck P . Common Knee Surgery does very little for some, Study Suggests, NY times - Health. http://www.nytimes.com/2013/12/26/ health/common-knee-surgery-does-very-little-for-some-study-suggests.html?pagewanted=all&_r=0. 2013

[17] King D . The Function of semilunar cartilages. J Bone Joint Surg Am. 1936; 1: 1069

[18] Longo UG , Loppini M , Forriol F , Romeo G , Maffulli N , Denaro V . Advances in meniscal tissue engineering. Stem Cells Int. 2012; 4203–46 10.1155/2012/420346 PMC3205710 25098366

[19] Scotti C , Hirschmann MT , Antinolfi P , Martin I , Peretti GM . Meniscus repair and regeneration: review on current methods and research potential. Eur Cell Mater. 2013; 26: 150–70 24057873 10.22203/ecm.v026a11

[20] Verdonk PCM , Van Laer MEE , Verdonk R . Meniscus Replacement: From Allograft to Tissue Engineering. Sports Orthop. Traumatol. 2008; 24(2): 78–82

[21] Sgaglione NA , Steadman JR , Shaffer B , Miller MD , Fu FH . Current concepts in meniscus surgery: resection to replacement. Arthroscopy. 2003; 19(1): 161–88 10.1016/j.arthro.2003.10.032 14673437

[22] Kohn D , Rudert M , Wirth CJ , Plitz W , Reiss G , Maschek H . Medial meniscus replacement by a fat pad autograft: An experimental study in sheep. International Orthopaedics (SICOT). 1997; 21: 232–8 10.1007/s002640050157 PMC3617697 9349959

[23] Kohn D . Autograft meniscus replacement: Experimental and clinical results. Knee Surg Sports Traumatol Arthrosc. 1993; 1(2): 123–5 8536007 10.1007/BF01565466

[24] Lubowitz JH , Verdonk PCM , Reid JB , Verdonk R . Meniscus allograft transplantation: a current concepts review. Knee Surg Sports Traumatol Arthrosc 2007; 15(5): 476–92 17333124 10.1007/s00167-006-0216-5

[25] Shelton WR . Meniscus Allograft Transplantation. Oper Tech Sports Med. 2007; 15(2): 81–5

[26] Buma P , Ramrattan NN , Van Tienen TG , Veth RPH . Tissue engineering of the meniscus. Biomaterials. 2004; 25(9): 1523–32 14697855 10.1016/s0142-9612(03)00499-x

[27] Ionescu LC , Lee GC , Huang KL , Mauck RL . Growth factor supplementation improves native and engineered meniscus repair *in vitro* . Acta Biomaterialia. 2012; 8: 3687–94 22698946 10.1016/j.actbio.2012.06.005 PMC3429724

[28] Centeno CJ , Busse D , Kisiday J , Keohan C , Freeman M , Karli D . Regeneration of meniscus cartilage in a knee treated with percutaneously implanted autologous mesenchymal stem cells. Med Hypotheses. 2008; 71(6): 900–8 18786777 10.1016/j.mehy.2008.06.042

[29] Madry H , Cucchiarini M , Terwilliger EF , Trippel SB . Recombinant adeno-associated virus vectors efficiently and persistently transduce chondrocytes in normal and osteoarthritic human articular cartilage. Hum Gene Ther. 2003; 14: 393–402 12659680 10.1089/104303403321208998

[30] Madry H , Cucchiarini M , Kaul G , Kohn D , Terwilliger EF , Trippel SB . Menisci are efficiently transduced by recombinant ade-no-associated virus vectors *in vitro* and *in vivo* . J Sports Med. 2004; 32: 1860–5 10.1177/0363546504265189 15572313

[31] Goto H , Shuler FD , Niyibizi C , Fu FH , Robbins PD , Evans CH . Gene therapy for meniscal injury: enhanced synthesis of proteoglycan and collagen by meniscal cells transduced with a TGFbeta(1) gene. Osteoarthr. Cartil. 2000; 8(4): 266–71 10903880 10.1053/joca.1999.0300

[32] Hidaka C , Ibarra C , Hannafin JA , Torzilli PA , Quitoriano M , Jen SS , *et al.* Formation of Vascularized meniscal Tissue by Combining Gene Therapy with Tissue Engineering. Tissue Eng Part A. 2002; 8(1): 93–105 10.1089/107632702753503090 11886658

[33] Stewart K , Pabbruwe M , Dickinson S , Sims T , Hollander AP , Chaudhuri JB . The effect of growth factor treatment on meniscal chondrocyte proliferation and differentiation on polyglycolic acid scaffolds. Tissue Eng. 2007; 13(2): 271–80 17504061 10.1089/ten.2006.0242

[34] Edwards SL , Mitchell W , Matthews JB , Ingham E , Russell SJ . Design of nonwoven scaffold structures for tissue engineering of the anterior cruciate ligament. AUTEX RES J. 2004; 4(2): 86–94

[35] Laurencin CT , Ambrosio AM , Borden MD , Cooper JA . Tissue engineering: orthopedic applications. Annu Rev Biomed Eng. 1999; 1: 19–46 11701481 10.1146/annurev.bioeng.1.1.19

[36] Dhandayuthapani B , Yoshida Y , Melawi T , Kumar DS . Polymeric Scaffolds in Tissue Engineering Application: A Review. Int J Polym Sci. 2011; 1–19

[37] Zaffagnini S , Giordano G , Vascellari A , Bruni D , Neri MP , Iacono F , *et al.* Arthroscopic collagen meniscus implant results at 6 to 8 years follow up. Knee Surg Sports Traumatol Arthrosc. 2007; 15(2): 175–83 16845545 10.1007/s00167-006-0144-4

[38] Bulgheroni P , Murena L , Ratti C , Bulgheroni E , Ronga M , Cherubino P . Follow-up of collagen meniscus implant patients: clinical, radiological, and magnetic resonance imaging results at 5 years. Knee. 2010; 17(3): 224–9. 19800801 10.1016/j.knee.2009.08.011

[39] Chiari C , Koller U , Dorotka R , Eder C , Plasenzotti R , Lang S , *et al.* A tissue engineering approach to meniscus regeneration in a sheep model. Osteoarthr Cartil. 2006; 14(10): 1056–65 16731009 10.1016/j.joca.2006.04.007

[40] Heijkants RGJC , Van Calck RV , De Groot JH , Pennings AJ , Schouten AG , Van Tienen TG , *et al.* Design, synthesis and properties of a degradable polyurethane scaffold for meniscus regeneration. Journal of Material Science: Materials in Medicine. 2004; 15(4): 423–7 10.1023/b:jmsm.0000021114.39595.1e 15332611

[41] Hannink G , Van Tienen TG , Schouten AJ , Buma P . Changes in articular cartilage after meniscectomy and meniscus replacement using a biodegradable porous polymer implant. Knee Surg Sports Traumatol Arthrosc. 2011; 19(3): 441–51 20802995 10.1007/s00167-010-1244-8 PMC3038217

[42] Van Tienen TG , Hannink G , Buma P . Meniscus replacement using synthetic materials. Clin Sports Med. 2009; 28(1): 143–56 19064171 10.1016/j.csm.2008.08.003

[43] Mueller SM , Shortkroff S , Schneider TO , Breinan HA , Yannas IV , Spector M . Meniscus cells seeded in type I and type II collagen — GAG matrices *in vitro* . Biomaterials. 1999; 20: 701–9 10353653 10.1016/s0142-9612(98)00189-6

[44] Yan LP , Oliveira JM , Oliveira AL , Caridade SG , Mano JF , Reis RL . Macro/microporous silk fibroin scaffolds with potential for articular cartilage and meniscus tissue engineering applications. Acta Biomater. 2012; 8(1): 289–301 22019518 10.1016/j.actbio.2011.09.037

[45] Ramakrishna S . Textile-based scaffolds for tissue engineering. Advanced Textiles for Wound Care. 2009; 289–321

[46] Hutmacher DW . Scaffold design and fabrication technologies for engineering tissues — state of the art and future perspectives. J Biomater Sci Polym Ed. 2001; 12(1): 107–24 11334185 10.1163/156856201744489

[47] Holloway JL , Lowman AM , Palmese GR . Mechanical evaluation of poly(vinyl alcohol)-based fibrous composites as biomaterials for meniscal tissue replacement. Acta Biomater. 2010; 6(12): 4716–24 20601243 10.1016/j.actbio.2010.06.025

[48] Holloway JL , Lowman AM , VanLandingham MR , Palmese GR . Interfacial optimization of fiber-reinforced hydrogel composites for soft fibrous tissue applications. Acta Biomater. 2014; 10(8): 3581–9 24814880 10.1016/j.actbio.2014.05.004

[49] El-Amin S , Kelly N , Pallotta N , Hammoud S , Lipman J , Ma Y , *et al.* Design and Evaluation of a Synthetic Fiber-Reinforced Hydrogel Meniscal Replacement. ORS Annual Meet. 2011; 417

[50] Gunja NJ , Athanasiou KA . Additive and synergistic effects of bFGF and hypoxia on leporine meniscus cell-seeded PLLA scaffolds. J Tissue Eng Regen Med. 2010; 4(2): 115–22 19937913 10.1002/term.221 PMC3553794

[51] Neves AA , Medcalf N , Smith M , Brindle KM . Evaluation of engineered meniscal cartilage constructs based on different scaffold geometries using magnetic resonance imaging and spectroscopy. Tissue Eng Part A. 2006; 12(1): 53–62 10.1089/ten.2006.12.53 16499442

[52] Chen G , Sato T , Ushida T , Hirochika R , Shirasaki Y , Ochiai N , Tateishi T . The use of a novel PLGA fiber/collagen composite web as a scaffold for engineering of articular cartilage tissue with adjustable thickness. J Biomed Mater Res A. 2003; 67(4): 1170–80 14624503 10.1002/jbm.a.10164

[53] Andrews SHJ , Ronsky JL , Rattner JB , Shrive NG , Jamniczky HA . An evaluation of meniscal collagenous structure using optical projection tomography. BMC Med Imaging. 2013; (13): 21 23879345 10.1186/1471-2342-13-21 PMC3726444

[54] Moutos FT , Freed LE , Guilak F . A biomimetic three-dimensional woven composite scaffold for functional tissue engineering of cartilage. Nat Mater. 2007; 6(2): 162–7 17237789 10.1038/nmat1822

[55] Moutos DPh , Guilak F (2009). Functional Properties of Cell-Seeded Three-Dimensionally Woven Poly (e -Caprolactone) Scaffolds for Cartilage. Tissue Eng Part A. 2009; 16(4): 1291–301 10.1089/ten.tea.2009.0480 PMC2862608 19903085

[56] Wood DJ , Minns RJ , Strover A . Replacement of the rabbit medial meniscus with a polyester-carbon fiber bioprosthesis. Biomaterials. 1990; 11(1): 13–6 2302444

[57] Marsano A , Wendt D , Raiteri R , Gottardi R , Stolz M , Wirz D , *et al.* Use of hydrodynamic forces to engineer cartilaginous tissues resembling the non-uniform structure and function of meniscus. Biomaterials. 2006; 27(35): 5927–34 16949667 10.1016/j.biomaterials.2006.08.020

[58] Kang SW , Son SM , Lee JS , Lee ES , Lee KY , Park SG , *et al.* Regeneration of whole meniscus using meniscal cells and polymer scaffolds in a rabbit total meniscectomy model. J Biomed Mater Res A. 2006; 77(4): 659–71 16514599 10.1002/jbm.a.30579

[59] Balint E , Gatt CJ , Dunn MG . Design and mechanical evaluation of a novel fiber-reinforced scaffold for meniscus replacement. J Biomed Mater Res A. 2012; 100(1): 195–202 22021218 10.1002/jbm.a.33260 PMC3222721

[60] Zur G , Linder-Ganz E , Elsner JJ , Shani J , Brenner O , Agar G , *et al.* Chondroprotective effects of a polycarbonate-urethane meniscal implant: histopathological results in a sheep model. Knee Surg Sports Traumatol Arthrosc. 2011; 19(2): 255–63 20635076 10.1007/s00167-010-1210-5

[61] Elsner J , Zur G , Guilak F , Linder-Ganz E , Shterling A . Design Optimization of a Polycarbonate-Urethane Meniscal Implant in the Sheep Knee. in 56th Annual Meeting of the Orthopaedic Research Society 2009

[62] Aufderheide AC , Athanasiou KA . Comparison of scaffolds and culture conditions for tissue engineering of the knee meniscus. Tissue Eng. 2005; 11(7): 1095–104 16144445 10.1089/ten.2005.11.1095

[63] Fisher MB , Henning EA , Söegaard N , Esterhai JL , Mauck RL . Organized nanofibrous scaffolds that mimic the macroscopic and microscopic architecture of the knee meniscus. Acta Biomater. 2013; 9(1): 4496–504 23085562 10.1016/j.actbio.2012.10.018 PMC3508287

[64] Baker BM , Shah RP , Huang AH , Mauck RL . Dynamic tensile loading improves the functional properties of mesenchymal stem cellladen nanofiber-based fibrocartilage. Tissue Eng Part A. 2011; 17(9-10): 1445–55 21247342 10.1089/ten.tea.2010.0535 PMC3079166

[65] Baker BM , Nathan AS , Huffman GR , Mauck RL . Tissue engineering with meniscus cells derived from surgical debris. Osteoarthr. Cartil. 2009; 17(3): 336–45 18848784 10.1016/j.joca.2008.08.001 PMC2672194

[66] Baker BM , Mauck RL . The effect of nanofiber alignment on the maturation of engineered meniscus constructs. Biomaterials. 2007; 28(11): 1967–77 17250888 10.1016/j.biomaterials.2007.01.004 PMC1847368

[67] Vrancken ACT , Buma P , Van Tienen TG . Synthetic meniscus replacement: a review. Int Orthop. 2013; 37(2): 291–9 23100123 10.1007/s00264-012-1682-7 PMC3560902

[68] Linke RD , Ulmer M , Imhoff AB . Replacement of the Meniscus with a Collagen Implant (CMI). Eur J Trauma Emerg Surg. 2007; 33(4): 435–40 26814740 10.1007/s00068-007-2188-7

[69] Buma P Total replacement of the meniscus-trammpolin.” [Online]. Available: http://www.bmm-program.nl/site/public/go/article.aspx7id=83&title=Artificial+meniscus. [Accessed: 14-Oct-2013]

[70] Verdonk R . Instructional Course Lecture: Alternative treatments for meniscal injuries. J Bone Joint Surg Am. 79-B(5): 866–73 10.1302/0301-620x.79b5.14193 9331051

[71] Petersen W , Tillmann B . Collagenous fibril texture of the human knee joint menisci. Anatomy and Embryology. 1998; 197(4): 31724 10.1007/s004290050141 9565324

[72] Mandal BB , Park SH , Gil ES , Kaplan DL . Multilayered silk scaffolds for meniscus tissue engineering. Biomaterials. 2011; 32(2): 639–51 20926132 10.1016/j.biomaterials.2010.08.115 PMC2991374

